# A Retrospective Cohort Study on the Clinical Characteristics of Patients with Surgical Blunt Bowel and/or Mesenteric Injuries among Motorcyclists and Car Occupants

**DOI:** 10.3390/healthcare10071323

**Published:** 2022-07-16

**Authors:** Ting-Min Hsieh, Po-Chun Chuang, Chun-Ting Liu, Bei-Yu Wu, Ching-Hua Hsieh

**Affiliations:** 1 Division of Trauma, Department of Surgery, Kaohsiung Chang Gung Memorial Hospital, Chang Gung University College of Medicine, 123 Ta Pei Road, Niao-Song District, Kaohsiung 833, Taiwan; hs168hs168@gmail.com; 2 Department of Emergency, Kaohsiung Chang Gung Memorial Hospital, Chang Gung University College of Medicine, 123 Ta Pei Road, Niao-Song District, Kaohsiung 833, Taiwan; bogy1102@cgmh.org.tw; 3 Department of Chinese Medicine, Kaohsiung Chang Gung Memorial Hospital, Chang Gung University College of Medicine, 123 Ta Pei Road, Niao-Song District, Kaohsiung 833, Taiwan; juntin0214@gmail.com (C.-T.L.); y7802@cgmh.org.tw (B.-Y.W.)

**Keywords:** surgical blunt bowel and/or mesenteric injuries (BBMIs), mortality, trauma

## Abstract

(1) Background: Surgical blunt bowel and/or mesenteric injuries (BBMIs) are rare but challenging for trauma surgeons. Surgical BBMI is associated with specific injury mechanisms, such as direct compression by the handlebar in motorcycle accidents or rapid acceleration and deceleration of the impact forces associated with seatbelt injuries in motor vehicle collisions. However, the discussions on the implications of BBMI and the mechanisms of road traffic accidents remain scarce. This retrospective study assessed the clinical and injury characteristics of surgically proven BBMI among motorcyclists and car occupants based on trauma-registered data obtained from a level I trauma center in Taiwan. (2) Methods: Medical data of 72 motorcyclists and 38 car occupants who had surgical BBMI between January 2009 and December 2020 were reviewed. Patient characteristics, injuries, and outcomes in both groups were compared and analyzed. (3) Results: Motorcyclists with surgical BBMI had a significantly higher Injury Severity Score (median [Q1–Q3], 18 (9–27) vs. 16 (9–18), *p* = 0.044) and lower Glasgow Coma Scale score (15 (11–15) vs. 15 (15–15), *p* = 0.034]) than car occupants. Motorcyclists with surgical BBMI had a higher incidence of pelvic fractures (18.1% vs. 2.6%, *p* = 0.032) and upper limb fractures (23.6% vs. 7.9%, *p* = 0.042) and a significantly higher rate of chest tube insertion than car occupants (29.2% vs. 10.5%, *p* = 0.027). However, there were no significant differences in the outcomes of morbidity and mortality between motorcyclists and car occupants with surgical BBMI. (4) Conclusions: This study demonstrated there were no significant differences in outcomes between motorcyclists and car occupants with surgical BBMI. However, motorcyclists with surgical BBMI were injured more severely, along with injuries to the head/neck and extremities, than car occupants.

## 1. Introduction

Blunt bowel and/or mesenteric injuries (BBMIs) are uncommon in the emergency department (ED) and account for approximately 3.1–4.7% of patients with blunt abdominal trauma (BAT) and 0.6–1.0% of trauma admissions [1,2,3]. BBMI is the third most vulnerable abdominal organ after the spleen and liver in the context of BAT. Even in the era when nonoperative management (NOM) is established to treat blunt hepatosplenic trauma [4], patients with surgical BBMI still need laparotomy. Therefore, acknowledging trauma patients with surgical BBMI remains an essential issue for trauma surgeons. In addition, given the low incidence and difficulty of diagnosis, familiarity with the entity can help provide medical aid to clinical physicians.

Although surgical BBMI is difficult to diagnose, it is associated with specific injury mechanisms, such as direct compression by the handlebar in motorcycle accidents or rapid acceleration and deceleration of the impact forces associated with seatbelt injuries in motor vehicle collisions [5,6]; thus, knowing the differences of the clinical characteristics of patients with surgical BBMI between motorcyclists and car occupants is an important issue. In Taiwan, motorcycle accidents account for most trauma injuries [7,8,9,10], while in Western countries, motor vehicle collisions contribute to most injury mechanisms in patients [11,12,13,14]. However, studies of BBMIs regarding the different injury mechanisms are scant in the literature. Furthermore, while the extant literature on BBMI has assessed the accuracy or effectiveness of imaging [2,15,16], risk factors of morbidity and mortality [17], and diagnostic delay [2,18,19], the implications of BBMI and road traffic accidents have not been explored extensively. This study aimed to investigate the differences in injury characteristics and outcomes of trauma patients with surgical BBMI between those motorcyclists and car occupants. This study was performed by retrospectively reviewing trauma-registered data obtained from a level I trauma center in Taiwan.

## 2. Materials and Methods

### 2.1. Ethical Approval

This study was approved by the Institutional Review Board (IRB) of Chang Gung Memorial Hospital (approval number 201902275B0). The requirement for informed consent was waived according to IRB regulations because of the retrospective study design.

### 2.2. Study Population

**Figure 1:**
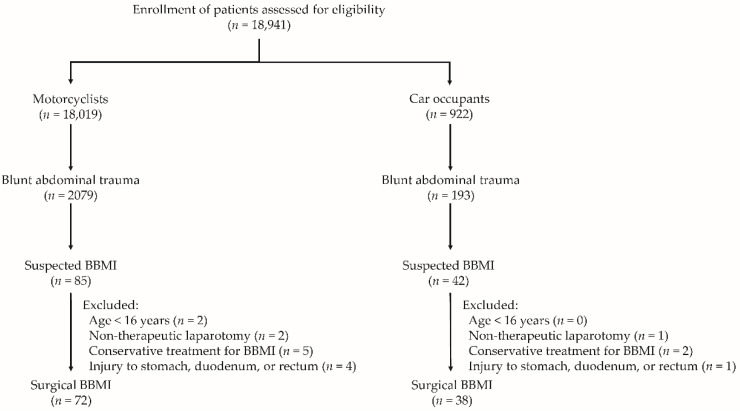
Flowchart depicting the enrollment of patients with surgical blunt bowel and/or mesentery injury (BBMI) according to the groups of motorcyclists and car occupants.

This study reviewed data from the Trauma Registry System from 1 January, 2009, to 31 December, 2020, in a 2686-bed level I trauma center that provides care to trauma patients in southern Taiwan [8,9,20]. All registered data recorded after trauma accidents were retrospectively collected and input into the hospital-based databank by two qualified nurses with specific responsibility for the integrity of the registered data [8,9,20]. Only motorcyclists and car occupants with BAT were included, whereas pedestrians involved in accidents or patients injured by causes other than motorcycle or car accidents were excluded. All patients (≥16 years of age) with surgical BBMI following BAT were surgically proven with small bowel, colon, or mesentery injuries. In total, 18,941 hospitalized patients were assessed. Among these, 18,019 motorcyclists and 922 car occupants were enrolled in this study (Figure 1). Of these, 2272 patients, including 2079 motorcyclists and 193 car occupants, had sustained BAT. There were 85 motorcyclists and 42 car occupants with suspected BBMI. After excluding those aged <16 years and who had received nontherapeutic laparotomy—conservative treatment for BBMI—and injury to the stomach, duodenum, or rectum during laparotomy, data from 72 motorcyclists and 38 car occupants who sustained surgical BBMI were eligible for further analysis.

### 2.3. Study Parameters

The data included information on age, sex, vital signs recorded at the ED, including systolic blood pressure (SBP), heart rate, respiratory rate, and Glasgow Coma Scale (GCS) score; trauma score, Injury Severity Score (ISS), Trauma-Injury Severity Score (TRISS), intubation at the ED, chest tube insertion, and unplanned intubation during hospitalization; operative findings, including isolated small bowel injury, isolated colon injury, isolated mesentery injury, and combined injury; and outcomes, including morbidity, multiple organ dysfunction, in-hospital mortality, 24 h mortality, bowel-related mortality, exsanguination-related mortality, length of ventilator use, and length of stay in the hospital and intensive care unit. The severity of injury in each body region was assessed using the Abbreviated Injury Scale (AIS), along with the presence of associated injuries.

### 2.4. Definitions

Surgical BBMI was defined as patients who had laparotomy-proven blunt bowel and/or mesentery injuries. Unplanned intubation was defined as incidental endotracheal tube insertion due to acute respiratory failure during hospitalization. Isolated bowel injury was defined as small bowel injury, including ischemia, rupture, serosa injury, or hematoma. Isolated colon injury was defined as only colon injury, including ischemia, rupture, serosa injury, or hematoma. Isolated mesenteric injury was defined as only mesentery injury, including ischemia, rupture, serosa injury, or hematoma. Combined injury was defined as either small bowel or colon injury concomitant with mesentery injury, including ischemia, rupture, serosa injury, or hematoma.

Morbidity information was obtained from chart records. Morbidity was defined as complications arising from acidosis (*n* = 33), sepsis (*n* = 21), pneumonia (*n* = 18), coagulopathy (platelet count < 150,000 mg/dL or international normalized ratio of prothrombin time or activated prothrombin time > 1.5 s, *n* = 47), septic shock (*n* = 8), urinary tract infection (*n* = 16), acute renal failure (*n* = 39), ventilator support (*n* = 27), intra-abdominal abscess (*n* = 11), bowel anastomotic leakage (*n* = 6), hyperbilirubinemia ≥ 2 mg/dL (*n* = 31), wound infection (*n* = 23), wound dehiscence (*n* = 7), pleural effusion requiring drainage (*n* = 18), stroke (*n* = 3), adult respiratory distress syndrome (*n* = 2), return to the operating room (*n* = 16), and renal function deterioration to hemodialysis (*n* = 1). Marshall et al. defined MODS as multiple organ dysfunction syndrome [21]. Bowel-related mortality was defined as mortality due to abdomen-related sepsis following surgery. Finally, exsanguination-related mortality was defined as mortality due to surgically proven hemorrhagic shock resulting from bowel or mesenteric bleeding.

### 2.5. Statistical Analysis

Collected data were compared using IBM SPSS Statistics for Windows (version 20.0; IBM Corp., Armonk, NY, USA). Continuous data were reported as medians and interquartile ranges, whereas categorical data were reported as frequencies and percentages. Two-sided Fisher’s exact or Pearson’s chi-square tests were used to compare categorical variables. Unpaired Student’s *t*-test was used to analyze normally distributed continuous variables, whereas the Mann–Whitney *U* test was used to compare non-normally distributed data. Statistical significance was set at *p* < 0.05.

## 3. Results

### 3.1. Clinical Characteristics and Outcomes of Motorcyclists and Car Occupants with Surgical BBMI

Cases of surgical BBMI in car occupants due to BAT were significantly higher than those in motorcyclists (19.7% [38/193] vs. 3.5% [72/2079], *p* < 0.001). Of the 110 trauma patients with surgical BBMI, 72 were motorcyclists, and 38 were car occupants (Table 1). There were no significant differences in age, sex, ED vital signs, Revised Trauma Scale score, or TRISS between the two groups. Motorcyclists had a significantly higher ISS (median [Q1–Q3], 18 [9–27] vs. 16 [9–18], *p* = 0.044) and lower GCS score (15 [11–15] vs. 15 [15–15], *p* = 0.034) than car occupants. In terms of management, significant differences in the rate of intubation at the ED and unplanned intubation during hospitalization were not found between the two groups. However, motorcyclists had a significantly higher chest tube insertion rate than car occupants (29.2% vs. 10.5%, *p* = 0.027). The operative findings revealed that car occupants had a higher rate of combined injuries (52.6% vs. 27.8%, *p* = 0.010) than motorcyclists. In addition, there were no significant differences in morbidity and mortality outcomes between motorcyclists and car occupants.

### 3.2. Injury Severity of Body Region of Motorcyclists and Car Occupants with Surgical BBMI

To investigate whether there was a different severe injury, defined as AIS ≥ 3, in various body regions between motorcyclists and car occupants with surgical BBMI, the injury severity in various body regions is summarized in Table 2. Motorcyclists had significantly higher rates of AIS head/neck ≥ 3 and AIS ≥ 3 than car occupants.

### 3.3. Associated Injuries to Motorcyclists and Car Occupants with Surgical BBMI

The injuries associated with surgical BMIs to motorcyclists and car occupants are summarized in Table 3. A significantly higher incidence of pelvic fractures (18.1% vs. 2.6%, *p* = 0.032) and upper limb fractures (23.6% vs. 7.9%, *p* = 0.042) was observed among motorcyclists than among car occupants, whereas no significant difference was found in the rate of associated injuries between the two groups.

## 4. Discussion

In this study, cases of surgical BBMI in admissions due to trauma injury (0.58%, 110/18,941) and after BAT (4.84%, 110/2272) among motorcyclists and car occupants were in accordance with previous reports [1,2,3]. However, although most patients with surgical BBMI were motorcyclists, patients of surgical BBMI among car occupants after BAT were significantly higher than motorcyclists (19.7% vs. 3.5%, *p* < 0.001). In addition, the ratios of surgical BBMI among motorcyclists and car occupants after BAT (3.5% and 19.7%, respectively) were also similar to those in our previous study on high-grade blunt hepatosplenic trauma between motorcyclists and car occupants (2.4% and 14.1%, respectively) [22]. Considering that motorcyclists account for 87% (whereas car occupants account for only 3.1%) of the trauma population associated with road transportation accidents in Taiwan [7], these abdominal injuries are more common among victims of car crashes [22,23] but not motorcycle accidents [10,24]. The risks of surgical BBMI among motorcyclists and car occupants were in accordance with the report by Raharimanantsoa et al. [13]. They utilized a scoring tool for predicting early detection of surgical BBMI and determined a threefold risk, wherein car accidents and motorcycle accidents were assigned 3 and 1 points, respectively, on the predictive score of surgical BBMI. This evidence further supports our study results, indicating that surgical BBMI occurs more commonly in car occupants than in motorcyclists. Moreover, it is worth mentioning that our data showed that there were more incidences of alcohol-intoxicated victims with surgical BBMI among car occupants than among motorcyclists. This result may also be in accordance with the report that in Taiwan, some drivers still did not adhere strictly to the regulations of compulsory seatbelt use and banning drunk driving [25,26]. Therefore, the diagnosis of surgical BBMI should not be neglected for trauma victims of car occupants.

Patients with surgical BBMI are accompanied by a high ISS. Bège et al. [14] reported that an ISS of >15 was an independent risk factor for predicting surgical BBMI. This study determined an average median ISS of 17 (9–25) on average for both populations. Injury severity was in accordance with data reported by Fakhry et al. (mean ISS: 16.7) [11] and Malinoski et al. (mean ISS: 17) [1]. In this study, motorcyclists had a significantly higher ISS than car occupants. Furthermore, motorcyclists had significantly higher rates of AIS head/neck ≥ 3 and AIS ≥ 3 than car occupants. The observed results are in accordance with those reports describing that motorcyclists are generally more likely to sustain injuries to the head and extremities [10,24] and the use of seatbelts can reduce the severity and rate of brain injuries due to car accidents [27]. This study revealed that motorcyclists with surgical BBMI had more severe injuries to the head/neck region with a lower GCS score. Although the incidence of intracerebral hemorrhage was not significantly different between the two groups, the number of cases with intracerebral hemorrhage was higher among motorcyclists (15.3%) than among car occupants (7.9%). During car crash accidents, the occupant’s lower extremities usually receive energy from the dashboard and sustain lower limb injuries at different levels [23]. Therefore, the higher occurrence of upper limb fractures, but not lower limb fractures, among motorcyclists than car occupants observed in this study was not surprising. Granieri et al. [28] conducted a retrospective study associated with motorcycle-related trauma and used multivariate analysis to highlight that besides age factors, the site of injury distribution should not be underestimated. They concluded that the risks of death for motorcyclists were twice in head trauma, 1.5 times in chest trauma, 1.3 times in abdomen trauma, and 1.2 times in pelvis trauma. Since patients with polytrauma could acquire BBMI [1,8,23], early recognition and adequate treatment of associated injuries in patients with BBMI following motorcycle and car traffic accidents could be important.

The occurrence of BBMI may be associated with pelvic fractures, indicating a high-energy impact. In a multicenter study, Kuper et al. [29] reported an incidence of 21.6% of concomitant abdominal trauma in 16,359 patients with pelvic fractures. Al-Hassani et al. [19] evaluated the predictors of the early diagnosis of surgical BBMI in 109 patients and concluded that pelvic fractures can significantly help in the early detection of BBMI. Johnson et al. [30] reported that patients with concomitant pelvic fractures and seatbelt signs below the anterior superior iliac spine on computed tomography (CT) had 32.43% abdominal injuries and 21.43% surgical abdomen. However, the reports published by Loftus et al. [31] were contradictory. They indicated that pelvic fracture was not an independent predictor of surgical BBMI in a multivariate analysis of 267 patients. In this study, there was a significantly higher incidence of pelvic fractures (18.1% vs. 2.6%, *p* = 0.032) among motorcyclists than among car occupants. However, surgical BBMI is more commonly observed among car occupants than among motorcyclists, implying that mechanisms other than pelvic fracture are associated with the occurrence of surgical BBMI. We believe that BBMI among car occupants would be caused by seatbelts, which are mostly associated with shearing force due to rapid acceleration and deceleration, whereas BBMI among motorcyclists would result from handlebar injuries or pelvic fractures associated with direct compression force. However, further evidence is required to support this hypothesis.

Surgical BBMI is easily ignored due to the low incidences in the era of desiring NOM for blunt visceral injuries, especially in patients who are intubated, unconscious, alcohol intoxicated, or have spinal cord injury due to unreliable physical examination. Malinoski et al. [1] considered that a diagnostic delay > 5 h and extra-abdominal AIS score ≥ 3 were independent risk factors for mortality in patients with surgical BBMI. Liao et al. [2] thought that delayed operation (>24 h) did not influence the prognosis of this trauma challenge, whereas ISS remained a significant risk factor for mortality. In the present study, although motorcyclists with surgical BBMI had more severe injury, they still had similar morbidity and mortality compared with car occupants. This better outcome may be attributed to the alert of trauma surgeons regarding this disease, experienced expertise in imaging studies of computed tomography, and improved medical care in our facility.

This study had some limitations. This study had a retrospective design with inherent selection bias and included a small number of patients from a center because of the low number of cases of surgical BBMI. Furthermore, the use of safety devices, including helmets, seatbelts, and airbags, during accidents is unknown. In addition, the use of anticoagulation treatment in trauma patients is unknown. Furthermore, the uniform impact of patient management and surgery by different physicians on the outcomes can only be assumed. In addition, given the lack of a protocol regarding the management of BBMI, the present study failed to obtain complete information about the CT examination with respect to its timing and protocol. However, although abdominal CT may be critical for diagnosing BBMI [32], this study only enrolled surgical BBMI patients who were proven surgically following laparotomy, thus excluding the possible ambiguous diagnosis that relies solely on imaging studies. Despite these limitations, this study may provide useful information on this rare disease called surgical BBMI following motorcycle and car traffic accidents.

## 5. Conclusions

This study demonstrated that motorcyclists with surgical BBMI were injured more severely and sustained injuries to the head/neck and extremities than car occupants with surgical BBMI. However, car occupants with surgical BBMI were more common. This finding suggests that the diagnosis of surgical BBMI should not be neglected in trauma victims, regardless of whether they are motorcyclists or car occupants.

## Figures and Tables

**Table 1:** Clinical characteristics and outcomes of motorcyclists and car occupants with surgical BBMI.

	Overall *n* = 110	Motorcyclists *n* = 72	Car Occupants *n* = 38	*p*-Value
Age, age	43 (28–57)	42.5 (24–61)	46 (38–55)	0.544
Male, *n* (%)	89 (80.9%)	55 (76.4%)	34 (89.5%)	0.097
ISS	17 (9–25)	18 (9–27)	16 (9–18)	0.044
RTS	7.84 (7.02–7.84)	7.84 (6.90–7.84)	7.84 (7.11–7.84)	0.686
TRISS	0.97 (0.92–0.99)	0.97 (0.90–0.99)	0.98 (0.93–0.99)	0.175
ED vital signs and conscious				
SBP (mm/Hg)	116 (87–137)	118 (94–139)	113 (86–130)	0.352
HR (beats/min)	100 (83–119)	101 (80–120)	99 (85–113)	0.952
RR (times/min)	20 (18–20)	20 (18–20)	20 (19–21)	0.325
GCS	15 (14–15)	15 (11–15)	15 (15–15)	0.034
Intubation at ED, *n* (%)	24 (21.8%)	19 (26.4%)	5 (13.2%)	0.110
Chest tube insertion, *n* (%)	25 (22.7%)	21 (29.2%)	4 (10.5%)	0.027
Unplanned intubation, *n* (%)	10 (9.1%)	6 (8.3%)	4 (10.5%)	0.735
Operative findings				
Isolated small bowel injury, *n* (%)	27 (24.5%)	18 (25%)	9 (23.7%)	0.879
Isolated colon injury, *n* (%)	10 (9.1%)	8 (11.1%)	2 (5.3%)	0.489
Isolated mesentery injury, *n* (%)	34 (30.9%)	26 (36.1%)	8 (21.1%)	0.104
Combined injury, *n* (%)	40 (36.4%)	20 (27.8%)	20 (52.6%)	0.010
Outcomes				
Morbidity, *n* (%)	77 (70%)	51 (70.8%)	26 (68.4%)	0.793
MODS, *n* (%)	59 (53.6%)	39 (54.2%)	20 (52.6%)	0.878
Mortality, *n* (%)	15 (13.6%)	9 (12.5%)	6 (15.8%)	0.633
24 h mortality, *n* (%)	5 (4.5%)	3 (4.2%)	2 (5.3%)	1.000
Bowel-related mortality, *n* (%)	3 (2.7%)	1 (1.4%)	2 (5.3%)	0.274
Exsanguination mortality, *n* (%)	8 (7.3%)	5 (6.9%)	3 (7.9%)	1.000
Use of ventilator (days)	0 (0–1)	0 (0–2)	0 (0–0)	0.234
ICU LOS (days)	3 (2–8)	3 (2–11)	3 (2–7)	0.934
Hospitalization LOS (days)	17 (11–31)	21 (12–35)	16 (11–29)	0.248

BBMI, blunt bowel and/or mesentery injury; ED, emergency department; GCS, Glasgow Coma Scale; HR, heart rate; ICU, intensive care unit; ISS, Injury Severity Scale; LOS, length of stay; MODS, multiple organ dysfunction; RR, respiratory rate; RTS, Revised Trauma Scale; SBP, systolic blood pressure; TRISS, Trauma-Injury Severity Scale. Data are presented as a number (percentage) or median IQR (25–75%).

**Table 2:** Severity of injury among motorcyclists and car occupants with surgical BBMI.

	Overall *n* = 110	Motorcyclists *n* = 72	Car Occupants *n* = 38	*p*-Value
AIS head/neck ≥ 3	11 (10.0%)	11 (15.3%)	0 (0.0%)	0.015
AIS face ≥ 3	0 (0.0%)	0 (0.0%)	0 (0.0%)	
AIS chest ≥ 3	28 (25.5%)	22 (30.6%)	6 (15.8%)	0.091
AIS abdomen ≥ 3	101 (91.8%)	64 (88.9%)	37 (97.4%)	0.159
AIS extremity ≥ 3	24 (21.8%)	20 (27.8%)	4 (10.5%)	0.037

AIS, Abbreviated Injury Scale; BBMI, blunt bowel and/or mesentery injury. Data are presented as number (percentage) or median IQR (25–75%).

**Table 3:** Associated injuries among motorcyclists and car occupants with surgical BBMI.

	Overall *n* = 110	Motorcyclists *n* = 72	Car Occupants *n* = 38	*p*-Value
Intracerebral hemorrhage	14 (12.7%)	11 (15.3%)	3 (7.9%)	0.372
Skull fracture	4 (3.6%)	4 (5.6%)	0 (0.0%)	0.296
Facial bone fracture	14 (12.7%)	12 (16.7%)	2 (5.3%)	0.132
Cervical spine fracture	2 (1.8%)	2 (2.8%)	0 (0.0%)	0.544
Clavicle fracture	7 (6.4%)	5 (6.9%)	2 (5.3%)	1.000
Scapula fracture	2 (1.8%)	1 (1.4%)	1 (2.6%)	1.000
Rib fracture	22 (20%)	14 (19.4%)	8 (21.1%)	0.841
Lung contusion	15 (13.6%)	9 (12.5%)	6 (15.8%)	0.633
Hemopneumothorax	22 (20.0%)	17 (23.6%)	5 (13.2%)	0.192
Spleen injury	8 (7.3%)	6 (8.3%)	2 (5.3%)	0.712
Liver injury	21 (19.1%)	12 (16.7%)	9 (23.7%)	0.373
Pancreas injury	6 (5.5%)	3 (4.2%)	3 (7.9%)	0.414
Kidney injury	7 (6.4%)	6 (8.3%)	1 (2.6%)	0.418
Diaphragm injury	3 (2.7%)	3 (4.2%)	0 (0.0%)	0.550
Great vessel injury	13 (11.8%)	9 (12.5%)	4 (10.5%)	1.000
Thoracic spine fracture	1 (0.9%)	1 (1.4%)	0 (0.0%)	1.000
Lumbar spine fracture	4 (3.6%)	2 (2.8%)	2 (5.3%)	0.607
Pelvic fracture	14 (12.7%)	13 (18.1%)	1 (2.6%)	0.032
Upper limb fracture	20 (18.2%)	17 (23.6%)	3 (7.9%)	0.042
Lower limb fracture	22 (20%)	16 (22.2%)	6 (15.8%)	0.423

Data are presented as a number (percentage).

## Data Availability

Not applicable.
